# Analysis and Advances in Additive Manufacturing as a New Technology to Make Polymer Injection Molds for World-Class Production Systems

**DOI:** 10.3390/polym14091646

**Published:** 2022-04-19

**Authors:** Adrian Benitez Lozano, Santiago Henao Álvarez, Carlos Vargas Isaza, Wilfredo Montealegre-Rubio

**Affiliations:** 1 Grupo de Investigación Calidad Metrología y Producción, Instituto Tecnológico Metropolitano, Medellín 050034, Colombia; santiagohenao@itm.edu.co (S.H.Á.); carlosvargas@itm.edu.co (C.V.I.); 2 Grupo de Investigación Diseño y Optimización Aplicada, Universidad Nacional de Colombia, Medellín 050034, Colombia; wmontealegrer@unal.edu.co

**Keywords:** mold additive manufacturing, polymer molds, subtractive manufacturing, mold characterization, rapid tooling, injection molding

## Abstract

The currently growing demand for metallic and polymeric products has undoubtedly changed the rules of manufacturing, enabling customers to more functionally define their products based on their needs. Nowadays, a new technique for rapid tooling, Additive Manufacturing (AM), can create customized products with more complex geometries and short life cycles (flexibility) in order to keep up with the new variables imposed by the manufacturing environment. In the last two decades, the migration from subtractive manufacturing to AM has materialized such products with reduced costs and cycle times. AM has been recently promoted to develop polymer molds for product manufacturing. This paper reviews the main findings in the literature concerning polymer molds created by AM compared to conventional (metal) molds obtained by subtractive manufacturing. Information about specific topics is scarce or nonexistent, for example, about the characterization of the most commonly injected materials and molds used in this type of technology, their mechanical properties (part and mold), designs for all types of geometries, and costs. These aspects are addressed in this literature review, highlighting the advantages of this alternative manufacturing process, which is considered a desirable technology worldwide.

## 1. Introduction

Over time, manufacturing industries have experienced more dynamic markets and growing competitiveness. Although Mold Additive Manufacturing (MAM) is found in high-impact scientific literature [1,2,3,4,5,6,7]. As a result, they need to be resilient in the face of quick changes in a market characterized by products with shorter lifecycles and great diversity in their manufacturing [8]. These changes have led to independence from conventional processes and a migration to mass production. More flexible marketing has resulted in lower-volume production with greater profitability because personalized products meet final customer requirements more precisely [9]. In addition, mass personalization has enabled a quick production of low-cost goods and services to satisfy customer needs [10], which requires flexibility and capacity to effectively respond to the demand. Additive Manufacturing (AM), a technology in line with the new requirements of global marketing, can be used for rapid tooling in order to develop high-quality products. As a result, in recent years, research into polymer injection mold design and rapid tooling by AM technologies has become more important because these innovative alternative technologies can help polymer industries achieve their objectives [11].

Previous studies [1,7,12,13,14,15,16,17] have demonstrated the potential of AM of polymer molds in the injection molding process and their impact, not only on time and cost reduction, but also on physical, mechanical, thermal, morphological, and other properties of the molded parts compared to those obtained with metal molds produced by conventional methods. Kampker et al. [1] studied the economic potential of different AM techniques with several materials to produce polymer tools, which were compared to their steel counterparts. With Selective Laser Sintering (SLS) and PA 3200 GF as mold material, they found a cost reduction of 84.2% compared to steel tools. Another study demonstrated the cost-benefit of integrating AM, using Digital Light Processing (DLP), into the conventional manufacturing process of injection molding to create mold inserts. It resulted in a cost reduction between 80% and 90% depending on the geometry of the mold insert developed for each product. In addition, a break-even point was established in [12] to determine how profitable AM is for Rapid Tooling (RT). In that case, the break-even points were 3400 and 500 for units with small and large geometries, respectively. Besides the economic aspect, another relevant field in AM is the study of the process and the characterization of the molded part and the mold obtained by different RT processes using AM. In the late 1990s, stereolithography (SLA), the first additive manufacturing technique, set a precedent in the production of injection molding tools. Authors such as Sadegh et al. [18] saw the viability of this type of mechanism to manufacture prototypes and small production series. Others delved into issues such as the capacity of the materials, the characterization of their mechanical properties, the post-treatment to increase the deflection temperature under load, and the efficiency of the manufactured tools in terms of molded parts [13,14].

In more recent studies, new AM techniques have been investigated. For instance, Triebs et al. [7] used two methodologies, i.e., PolyJet and SLS, with mold inserts created employing digital ABS and PA 3200 GF, respectively. They observed a mechanical difference in the molded parts, which was apparently due to the poor thermal conductivity and increased roughness of the polymer molds compared to their aluminum counterparts. Additionally, they discussed the crystallinity of the molded part made of polypropylene (PP) and how nucleating agents favored the crystallization rate. Another study analyzed the thermal, mechanical, and thermo-mechanical properties of epoxy-based PolyJet molds to produce small series of PLA parts [15]. Other authors have examined issues related to failure over the lifespan of the molds and established diagnoses based on their findings [16,17]. Polymer research has analyzed the thermal, mechanical, and rheological characteristics of these materials. For example, a study [19] investigated the effects of process parameters on the strength and fatigue behavior of 3D printed PLA-graphene. Its experimental results indicate that fatigue lifetime clearly depends on process parameters, as well as loading amplitude and frequency. In Fused Filament Fabrication (FFF), heat transfer plays a particular role and determines the temperature history of the merging filaments; in turn, the in-process monitoring of the temperature profile guarantees the optimization and thus the improvement of interlayer adhesion [20]. This is very important to ensure the best quality of the piece.

This article presents a comprehensive literature review of the main findings in recent research into AM (as an alternative to obtain molds for injection molding processes), a comparative analysis between AM and subtractive technologies, and research topics that should be further addressed. Section 2 below introduces the subject, the chronology of conventional manufacturing and additive manufacturing for injection molds, the state of the art, and the research approach of this paper. Subsequently, Section 3 describes the methodology of this systematic literature review and a bibliometric analysis. Section 4 details the latest techniques and guidelines applied to mold design. Section 5 deals with the characterization and performance (mechanical properties) of the materials used in AM. Section 6 discusses cost evaluation. Finally, Section 7 draws the conclusions.

## 2. Chronology of Conventional Manufacturing vs. Additive Mold Manufacturing

**Figure 1:**
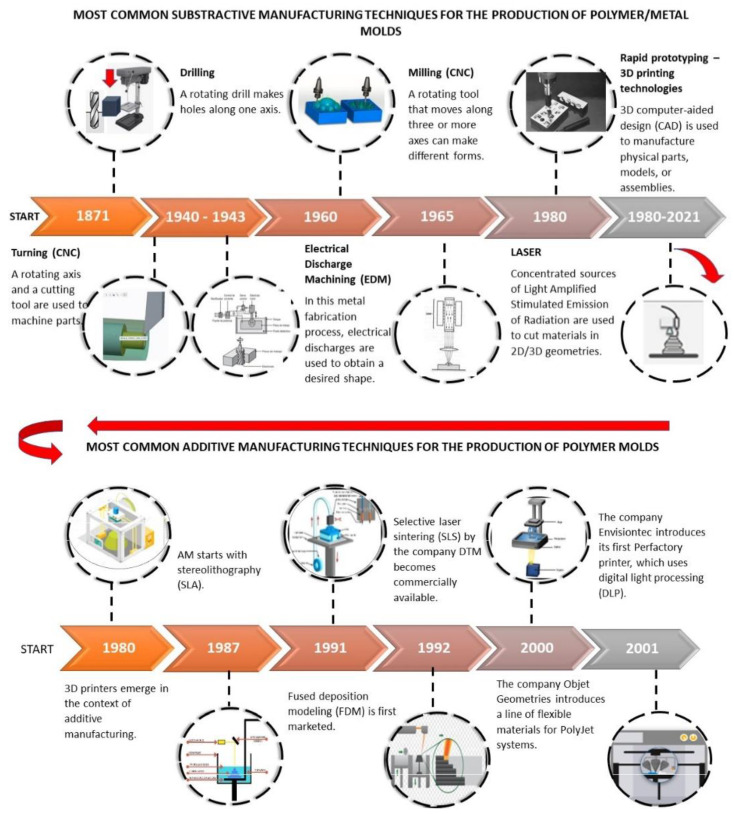
Chronology of the most common subtractive and additive manufacturing techniques for polymer/metal molds.

As shown in Figure 1, subtractive manufacturing dates back to 1871, with the development of the drill press with tools to make holes, nuts, tube flaring, and countersinks, which are essential for conventional cooling channels and fasteners in the mold industry. Later, between 1940 and 1943, the first machining operations supported by Computer Numerical Control (CNC) were developed. Subsequently, in the 1960s, this technology was extended to conventional milling, a fundamental process in the conventional mold industry for metal and polymer materials. Between 1965 and 1980, advanced machining processes were developed, e.g., Electro Discharge Machining (EDM) and LASER (1980). Such processes were very useful for detailing and finishing, generally, mold cavities and vents to release the pressures generated when the molten material is compressed. Since the 1980s, there has been a “boom” in additive mold manufacturing and its variants, which are described in Figure 1 (bottom). Different additive manufacturing techniques have paved the way for the production of polymer molds, and, although they are very different in principle and execution, they have achieved significant results for this type of applications. Figure 1 shows the chronology of additive manufacturing techniques used to produce polymer molds that are commonly found in the literature. The years mark the period of commercialization of each technique [17,18,19,20,21,22].

In 1980, the term 3D printing was introduced by Hideo Kodama, who invented the single-beam laser method that opened the door to the development of new 3D printing equipment and patents. In 1987, Charles W. Hull invented the first 3D printing equipment, called SLA-1, which used a technique known as stereolithography. In this technique, a photopolymer contained in a vat undergoes solidification produced by a laser, which is aimed at the cross section of the piece and gradually descends on the z-plane depending on the specified height. In 1991, the company Stratasys commercialized the first technique to extrude materials in the form of a filament; it was called Fused Deposition Modeling (FDM). In this technique, the material is melted using hot runners, which extrude the material layer by layer. In 1992, a new AM technique known as Selective Laser Sintering (SLS) entered the market. In it, the material, in powder form, is selectively sintered by a high-power CO_2_ laser beam onto the cross-section of the model. The first commercially available 3D printing system, called PolyJet, was launched by the company Objet Geometries in 2000. This system uses a jetting head to inject a UV-sensitive liquid resin that solidifies on a platform until the desired object is obtained.

In 2001, Digital Light Processing (DLP) technology, developed by Texas Instruments in the field of projectors, was introduced by the company Envisiontec at the EuroMold (a trade fair for moldmaking). In DLP, multiple micromirrors reflect a light source onto the printing material contained in a vat, which is then solidified layer by layer until the part is obtained.

Thus far, many studies have investigated the performance of these techniques for injection processes because they offer alternatives to meet the new needs of the market. More specifically, the behavior and performance of PolyJet 3D printing for RT applications have been some of the most widely studied. This technique produces high-performance tools in terms of thermal and mechanical properties thanks to its multi-material technology and high resolution, which ensure a good surface finish [18,23].

## 3. Methodology

**Figure 2:**
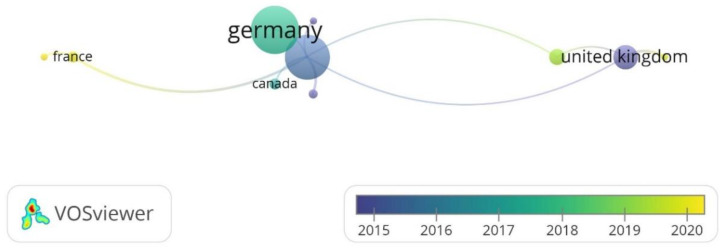
Network of countries based on co-authorship.

**Figure 3:**
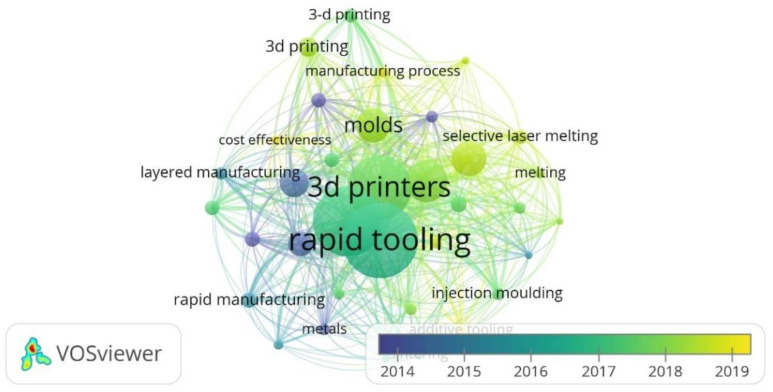
Network of keywords retrieved from the literature search.

The most important concepts in the field of AM were used here to conduct an exhaustive search and collect information. The initial keywords were “Additive Manufacturing”, “Rapid Tooling”, “Injection molding”, “cost”, “Failure”, “Polymer Mold”, and other terms that fall within the scope of this review. The Scopus and ScienceDirect databases were used for this purpose because they compile a considerable amount of world-class information in different research fields. This process was complemented with a more general search on the topic using the Scopus database, which was selected because of its comprehensiveness in terms of information, abstracts, and citations. Similar terms were refined using Science Direct Topics to obtain an adequate string of keywords. A bibliometric analysis and networks were used to examine and understand trends in this field in terms of authors and countries (Figure 2 and Figure 3).

A search string with the keywords above was used in the Scopus reference database, including Boolean operators to narrow down or filter the results as described by Burnham 2006 [23]. Once the strings shown in Table 1 were obtained, filters were used to exclude terms such as “3D printer” or “manufacture”. The search was limited to documents published between 2013 and 2021, and “Rapid tooling” was taken as the key term because it is articulated with the other concepts in this review. After conducting the advanced search with each string, the list of references in the fourth column in Table 1 was compiled. These are the documents reviewed in this paper.

Subsequently, a general search string was used in one of the reference databases (Scopus), and the results were exported to carry out a bibliometric analysis implementing VOSviewer software (version 1.6.16). The latter was employed to create networks of scientific publications, scientific journals, researchers, research organizations, countries, keywords, and terms [24,25] in order to understand current trends in the field analyzed in this review.

**Table 1:** Search results obtained with each string.

TITLE-ABS-KEY	Number of Retrieved Documents and Related References		
	Without Filter	Filter	References
“Additive manufacturing” AND “Rapid tooling” OR “Polymer Mould”	81	26	[1,2,3,4,5,6,7,8,9,10,11,12,13,14,15,16,17,18,19,20,21,22,26,27,28,29]
“Rapid tooling” AND “Additive manufacturing ”AND “rapid manufacturing” OR “Cost model” OR “Cost Advantage” OR “Cost analysis” OR “production economics” OR “3D printing” OR “cost estimation models” OR “Injection moulding”	83	11	[1,30,31,32,33,34,35,36,37,38,39]
“Additive manufacturing” AND “Rapid tooling” AND “Injection molding” AND “Failure”	46	31	[3,4,5,6,7,8,9,10,11,12,13,14,15,16,17,18,19,20,21,22,23,24,25,26,27,28,29,30,31,40,41,42]
“Additive manufacturing” AND “Rapid tooling” AND “Injection molding” AND “Design”	15	12	[1,21,23,24,25,28,29,40,43,44,45,46]
Filter: Review of the abstract and relationship with the search string			

### Bibliometric Analysis

As mentioned above, VOSviewer software (version 1.6.16) [13,14] was used to carry out a bibliometric analysis. This software was employed to construct networks, analyze the metadata, and establish relationships between the results of the following search string: TITTLE-ABS KEY “Additive manufacturing” AND “Rapid tooling” OR “Polymer Mold”. In this case, the keyword “Rapid Tooling” was limited to publications between 2013 and 2021. The bibliographic database was exported from Scopus to create, visualize, and explore three networks of great interest for this review.

Figure 2 shows the first network, which connects countries based on co-authorship. VOSviewer was configured so that the minimum number of documents per country was 3, which resulted in a network of 11 out of the 25 countries in the bibliographic references. India, Malaysia, New Zealand, Romania, and Spain were filtered out because the total strength of their links was not significant for this review, and they did not contribute relevant information to this analysis. Figure 2 is a network of keywords represented by labeled circles, where the more weight the item has, the bigger the label and the circle. The country with the highest weight is Germany because it presents the most abundant scientific production concerning Rapid Tooling (21 documents). Countries such as India and Spain have contributed a considerable amount of scientific production (10 and 5 articles, respectively); however, in this bibliometric analysis, they are not especially relevant because they do not have a strong relationship of co-authorship with other countries. The United States has the highest level of co-authorship among the 5 countries in Figure 1, and the strongest co-authorship relationship is that between Italy and the United Kingdom, where Additive Manufacturing and Rapid Tooling have been recently explored in depth.

The links established by co-occurrences of keywords were also analyzed. The minimum number of co-occurrences of keywords was set to 5. Among the 1413 keywords in the bibliographic references, 35 were above this threshold. In this case, no keywords were eliminated to construct the network. In Figure 3, the most prominent elements in the network are the keywords “Rapid Tooling” (102 occurrences) and “Additive Manufacturing” (93 occurrences), as expected. Likewise, term “Rapid Tooling” presents a strong connection with all the keywords retrieved from the literature search.

## 4. Mold Design

Some of the main issues in mold injection processes are efficient material processing and obtaining products at reasonable prices that reflect a strong economy of scale [30]. Mold design and the simulation of this process are essential aspects in the product life cycle [1], quality assessment, viability, and productivity of parts manufactured by injection.

**Figure 4:**
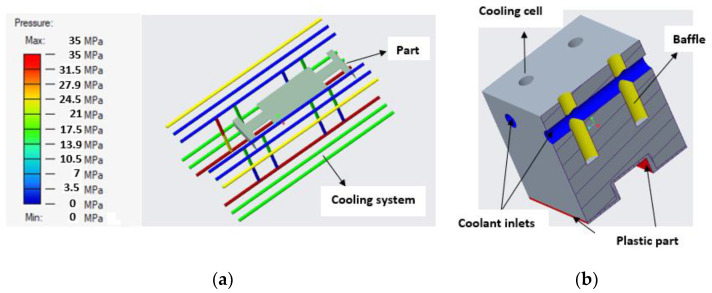
(**a**) Pressure field and temperatures in a refrigeration system. (**b**) Cooling system by means of baffles.

Generally, mold design is one of the most important aspects in the product life cycle because it determines the quality, viability, and productivity of parts. Mold design is necessary because parts should meet specific requirements, and, for that purpose, it is fundamental to know some characteristics of the piece to be manufactured, such as its geometry, weight, material, and volume [31]. Several mold design practices based on scientific findings represent benchmarks or references for recent research in this area. Currently, molds are designed with efficient cooling systems, air vents, and cooling channels that shorten the cycle time of injection molding processes, as shown in Figure 4. Many authors have adopted methodologies based on genetic algorithms to achieve efficiency in cooling systems that release the air trapped in injection molds, thus improving the quality, heat transfer, channel geometry, and formability of the injected product [32,33,34,35].

**Figure 5:**
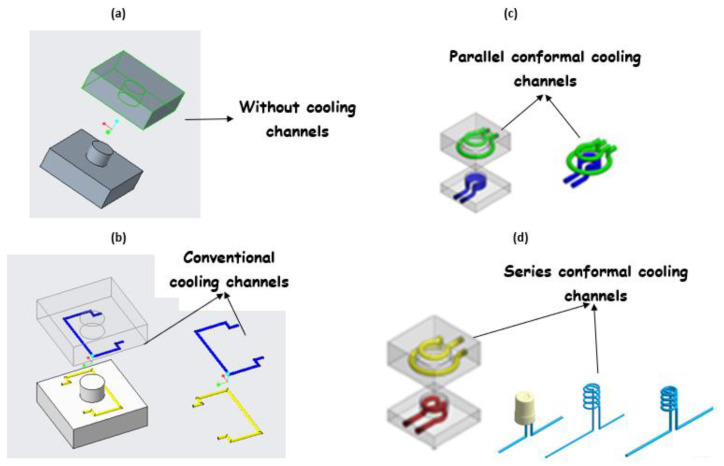
Solid models of cavity insert and cores (**a**) without cooling channels, (**b**) with conventional cooling channels, (**c**) with parallel conformal cooling channels, and (**d**) with series conformal cooling channels.

In addition, rapid prototyping technologies have been applied to manufacture molds with different types of low-pressure cooling channels for materials such as wax. Recent research in this area has focused on reducing cooling times [34]. Figure 5 compares the cooling performance of four injection molds with different cooling channels. Series conformal cooling channels (Figure 5d) are highly recommended in [34,43] to reduce the cooling time during the process because their cooling efficiency is approximately 90%.

Mold design should observe the guidelines and best practices of traditional injection molds. These design concepts can be applied to PolyJet molds, but alterations are required to compensate for the mechanical, thermal, and dimensional characteristics of plastic molds [40,43,44]. Conformal cooling channels show great potential for substituting conventional straight-drilled cooling channels because they can provide more uniform and efficient cooling effects, and thus improve the production quality and efficiency significantly [45]. Table 2 presents a technical guide to design mold cavities.

## 5. Performance and Properties of Mold Materials and Injected Polymers

Molds or inserts used in injection molding processes can be produced by additive manufacturing, which is referred to here as Rapid Tooling for Injection Molding (abbreviated as RTIM in this paper) [47,48]. Currently, RTIM using polymeric materials is being explored thanks to the development of additive technologies for polymers, greater access to these additive technologies, and their lower costs compared to metal additive technologies [49,50,51]. RTIM has thus produced a new market niche in injection molding by enabling low-volume production.

Polymer RTIM poses several challenges regarding its performance and effects on the properties of the injected parts made of polymers. The performance of polymer RTIM (intended for low-volume production) has been compared to that of traditional metal molds in terms of useful life, mechanical and thermal behavior, and other characteristics; nevertheless, their performance is completely different.

Most studies into polymer RTIM have focused on the performance of the mold and the properties of the injected parts, two elements that will be discussed below.

### 5.1. Failures in Polymer RTIM

**Figure 6:**
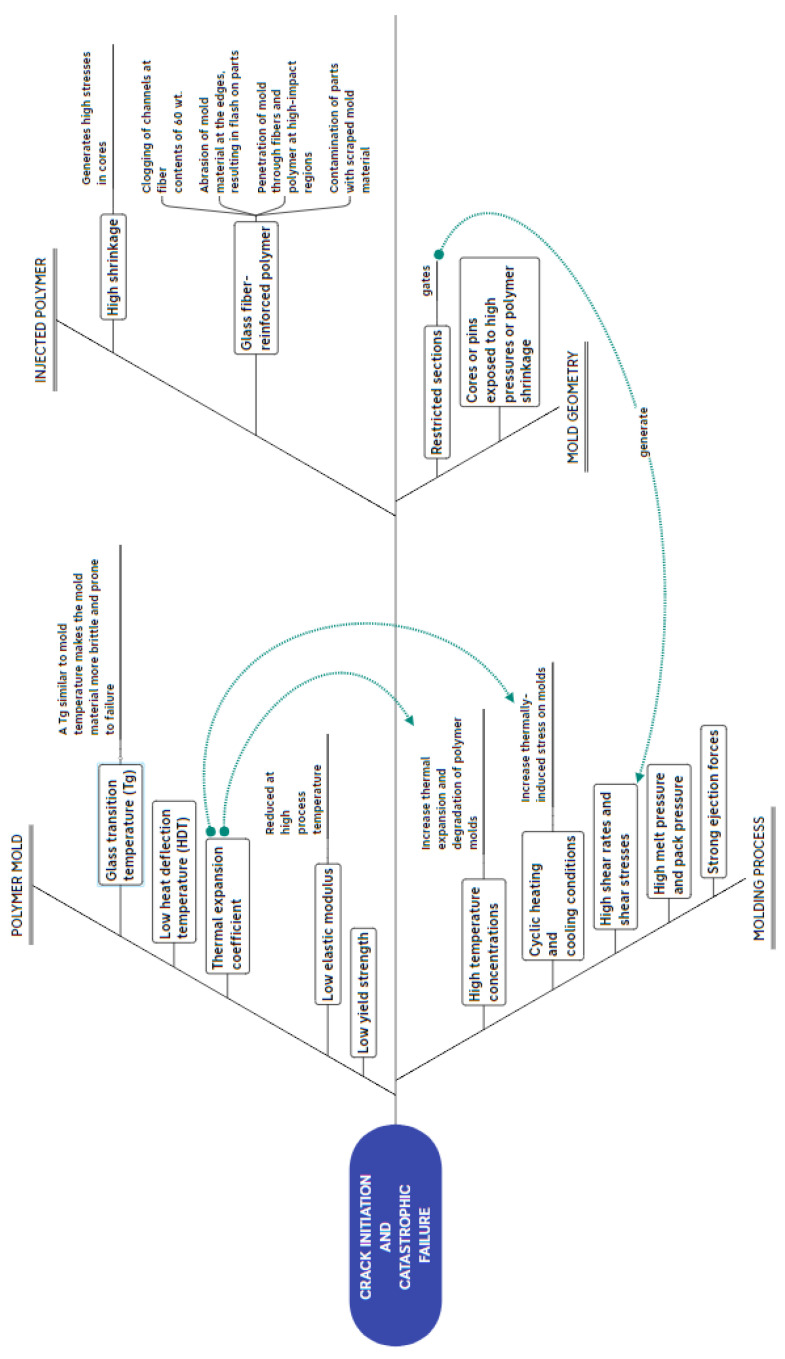
Ishikawa diagram of different sources of crack initiation and catastrophic failure in polymer RTIM.

Failures in polymer RTIM can occur due to several factors derived from the material of the polymer mold (i.e., glass transition temperature [5,52], heat deflection temperature [1,53], thermal expansion coefficient [6,54], and its mechanical properties [3,41,55,56]); the high shrinkage of the injected polymer [41,52] or the use of fiber-loaded materials [42]; the conditions of the injection process at high injection temperatures [3,57]; the heating and cooling cycle of the process [47,54]; extreme conditions of high shear stress, shear strength, and pressures during injection; strong part ejection forces [3,6,41,58]. Mold geometry can also contribute to failures in very specific sections, such as injection points and thin mold cores or pins that are weakened when subjected to high pressures or contractions of the injected material [3,52,58]. Some of these factors may be more critical than others, or they can produce a combined effect. The Ishikawa diagram (cause-effect diagram) in Figure 6 connects details and relates different sources of crack generation and propagation in polymer RTIM that lead to subsequent failures.

### 5.2. Characterization of Properties of Polymer RTIM and Injected Materials

In order to extend the lifetime of polymer RTIM products, it is important to find a balance between mechanical properties, thermal properties, and injection molding process conditions for a given mold. Additionally, the injected material is affected by the characteristics and properties of the mold, as well as the conditions of the injection process. Table 3 summarizes studies that have evaluated different polymer RTIM processes and their respective injected materials. This table also includes a characterization of the properties of the mold material, the injected part, and the method adopted to evaluate the injection process (i.e., predicted by computer simulations or monitored experimentally by sensors and/or data collection equipment).

## 6. Previous Studies of the Cost Model

In recent decades, in the context of the new industrial revolution, the technological potential of AM has increased and favored the development of different technological enablers such as cloud computing, cyber-manufacturing, and augmented reality [73]. This presents an ideal scenario for the creation of intelligent companies with a high degree of efficiency in their processes. However, during this technological advancement, the field of AM has been slow in establishing accurate cost models that can support corporate decision-making. Current literature describes different cost models classified by approach, AM technique, or the field of application where they are evaluated [74,75,76,77]. As this study is focused on AM in Injection Molding (IM), the following subsections highlight the main findings and results of cost models that have been used in this area.

### 6.1. Cost Models for AM as a Disruptive Technology in the IM Process

According to the literature, AM has been established as a disruptive technology that seeks to replace traditional manufacturing (TM) [36] because, compared to many conventional approaches, AM offers design freedom to manufacture complex and integrated parts. Using AM, tools or other processes are not necessary to create functional parts; hence, AM reduces the time needed to introduce a product into a market and, consequently, its total costs [36]. Many studies have compared and evaluated the break-even points of cost and production times of AM and IM for certain lot sizes. For example, Hopkinson and Dickens [37] were some of the first authors who identified the main sources of costs of Rapid Manufacturing (RM) related to AM. They showed that AM can compete against IM costs in situations of relatively high volumes. In the analysis and cost model they proposed, machinery, labor, and material costs represent the most critical variables [37]. Other authors later expanded on the work of Hopkinson and Dickens because they did not take into account a series of considerations in their model. For example, subsequent studies have investigated the construction and orientation of the manufactured parts (where packaging and distribution also play an important role), recycled material, and direct and indirect costs. Additionally, other papers have analyzed the production of copies of the same part and simultaneous production of different parts by SLS [38,39].

Another study [78] aimed to reduce costs and the final redesign of a part of a component previously produced by IM. In said study, it was demonstrated that RM can have economic potential for medium-sized production lots, and a break-even point was found at a production volume of around 87,000 units. However, a critical point in the AM model was the high acquisition cost of the SLS systems, which can be a decisive factor to migrate from one technology to the other [78]. Moreover, as a result of the growing popularization of low-cost 3D printing, additive techniques have had an exponential evolution. In 2015, $4.2 billion dollars were spent on AM in the US [79], which demonstrates the expansion of these alternative manufacturing techniques.

Achillas et al. [80] furthered the work of Hopkinson and Dickens because they not only evaluated the costs associated with SLA, SLS, and FDM techniques, but also included in their model the emerging PolyJet technique. The latter was used as an RT manufacturing method whose process was complementary rather than disruptive to the IM process. In their study, the key parameters were determined by lead time and total production cost. They also included variables such as time and pre-processing and post-processing cost. In their case, using RT to make soft molds by means of PolyJet was a very cost-effective method to manufacture new products in the range from 100 to 1000 units, while SLS was the most cost-effective AM technology in terms of time and cost.

In [79], the authors calculated the break-even points of AM and TM as a function of part mass, density, and lot size. Additionally, they took into account the cost of the material, equipment purchase, initial capital cost, time constraints, waste, overhead costs, etc. in order to construct a complete and realistic model. Based on this, they carried out a sensitivity analysis that showed that, in AM, material cost and part density were the variables most susceptible to variation; in turn, in IM, material cost, and mold cost per part presented the greatest changes. A lot size of approximately 200 units was the break-even point when deciding between AM and IM [79].

### 6.2. Cost Model as a Complement to AM in IM

Recently, from the perspective of cost estimation, some studies have investigated how AM can create added value when it is used as a complement to the IM process. Nevertheless, there is still a gap in the literature concerning the economic aspect of this object of study; hence, the following paragraphs will detail some articles that have encouraged the combination of these two technologies.

In [12], the authors sought to create synergy between the AM technique called Digital Light Processing (DLP) and conventional manufacturing processes. In their study, the main costs were pre-processing, construction, material, post-processing, and overhead. Regarding IM, the variables that most contributed to the cost were mold, material, and production. They concluded that tool cost was decreased by 80% when AM was used instead of CNC to create molds. The reduction in tooling cost could be approximately € 3489 (US$ 3995) for the largest geometry and € 996 (US$ 1140) for the smallest geometry in their study. Later [74], the same authors continued to study the cost estimation model employing the same AM technique, finding break-even points of up to 110,000 pieces when RT was used in IM. In addition, they reported longer processing times in AM (increasing the processing cost by 4%) because a longer cooling time was needed for polymer molds [74].

Kampker et al. [1] made a technological and economic comparison of 10 AM materials in the context of RT in order to provide guidelines to select materials for this type of applications. They found that the mold material with the greatest potential was PA3200 GF using the SLS technique, which reduced costs by 84.2% compared to tool steel. A year later, Ayvaz et al. [81] created an extended model to estimate costs and lead times of AM tools in IM. In their model, tool life was a key variable. They concluded that, using AM, tooling costs, by an estimated 20% to 66%; lead time, by up to 50%.

**Figure 7:**
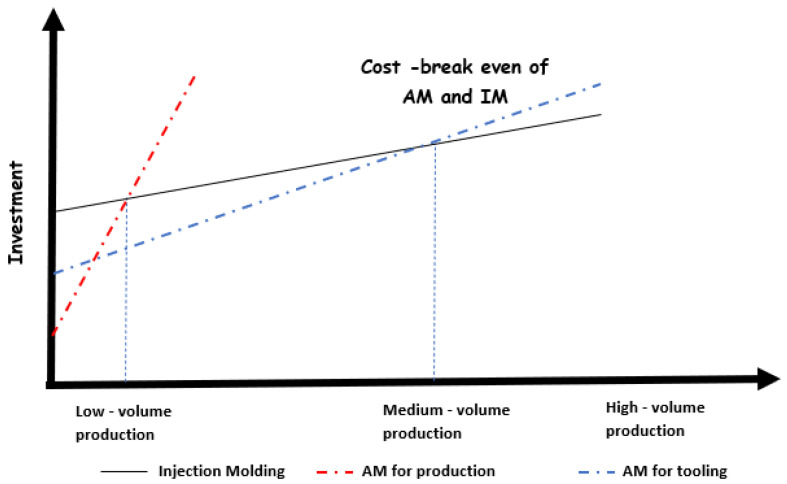
Cost curves of IM, AM for production, and AM for tooling.

Figure 7 shows the investments needed if AM complements IM for soft tooling and if AM replaces IM to manufacture functional parts. As AM has become more widespread in the last decade, companies such as MakerBot Inc. and Ultimaker Inc. have made parts at lower costs [82]. Nowadays, 3D printers are more affordable due to a decrease in the cost of computer processors and the expiration of patents that protected existing systems [79]. In Figure 7, the investment needed to produce units by AM is very low when production volumes are low; however, as the number of units increases, the investment is drastically affected. This is due to the longer processing and post-processing times required to improve the final properties of parts made by AM. In addition, because the raw material of AM is usually up to 10 times more expensive than that of IM [79], the break-even point of AM is found at low-volume production [74]. Nevertheless, IM represents a very high initial investment, partly due to the cost of the tooling. Although the production cost of a few parts by IM is relatively low, other alternatives could reduce it. For instance, using soft tooling by AM for IM would require a medium-sized investment and would be suitable for low- and medium-volume production, as shown in Figure 7.

Table 4 summarizes studies that have estimated the costs of AM as a disruptive and complementary technology to IM.

## 7. Conclusions

Mold design, an especially relevant aspect of the mold life cycle, represents around 80% of the total production cost. Therefore, designers should analyze and study technical guides (such as that in Section 4) in detail to properly design complete structures (e.g., ejection systems or ejectors, guides, cavities, runners, and gates).

Conformal cooling channels represent an innovative technique in mold design because they achieve shorter cycle times than conventional and parametric cooling channels. This technique should be further explored because it is closely related to AM.

Emerging technologies such as AM meet the new requirements imposed by the market (e.g., personalization and reduction of the product life cycle) because the products are made faster and complex parts can be freely designed. However, the costs associated with this type of technology increase exponentially because the materials, the acquisition of the machine, and processing time can affect the profitability of the process chain. Therefore, recent research has focused on the creation of cost models where AM is complemented by TM in the context of IM for low-, medium-, and high-volume production, which generates new business models and improves the efficiency of the processes.

The thermal and mechanical performance of polymer molds made by additive manufacturing for polymer injection is completely different from that of traditional metal molds. Hence, polymer molds should be previously evaluated to estimate their durability and changes caused by the injection process conditions. It is also important to quantify the properties of the polymer mold material and how they can affect the quality characteristics of the injected part, such as dimensional accuracy, shrinkage, and defects. This evaluation and economic manufacturing criteria can be used to justify the use of polymer injection molds made by additive manufacturing, which are generally well suited for medium- and low-volume production.

## Figures and Tables

**Table 2:** Technical guide to design mold cavities based on recent studies in the field [40,43,44,45,46].

**Mold Cavities**		
Draft	Use angles of approximately 3–5 degrees for the vertical wall. This will reduce mold damage, and the formed parts are less likely to resist ejection from the mold.	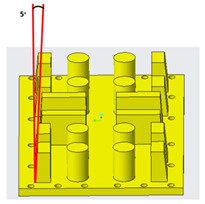
Parting surfaces	Make sure that parting surfaces have minimal flash. For this purpose, try to efficiently adjust the clamping force to compress the plastic material. Check injection parameters such as injection rate, temperature, and pressure.	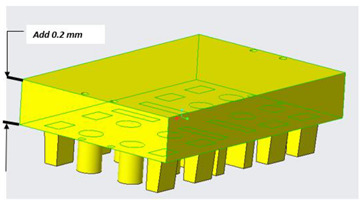
Core pins	Use core pins with an aspect ratio of 3:1 (height: width). Core pins could deflect due to the pressure in the filling process. A 3D printed insert can be designed to improve mold longevity.	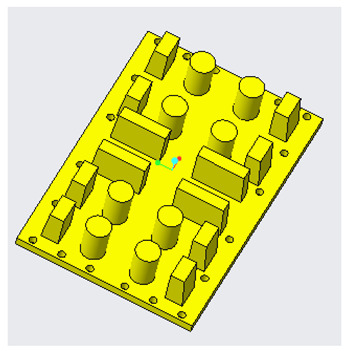
Holes	Use holes with diameters larger than 0.8 mm. Smaller holes could be machined prior to mold assembly.	
Shrinkage compensation	It is important to know the contraction or expansion of the printed material, generally in percentages. Based on these data, scale the core and the cavity to compensate for the shrinkage of the resin that occurs with conventional injection molding.	
**Mold Components**		
Gates	Enlarge the gates depending on the viscosity of the plastic material used for the part and the mold’s flow characteristics. Use or design gates three times larger than those used in metal molds. Make edge gate thickness equal to the wall thickness of the part at the point of injection. These measures will improve material flow and decrease pressure within the tool.	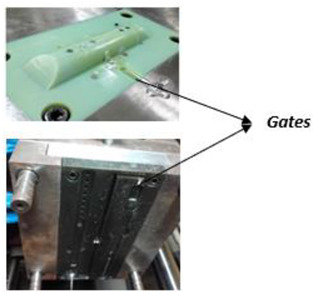
Runners	Hot runner systems are not recommended. If they are used, they do not require adjustment.	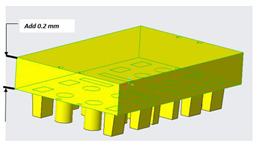
Sprue	Use a sprue bushing with a minimum draft angle of 3 degrees. If a bushing is used, undersize the hole by 0.2–0.3 mm before printing and ream to size during mold assembly. Avoid physical or direct contact between the molding machine’s nozzle and the mold insert.	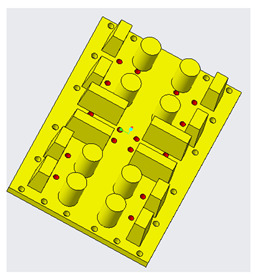
Ejection system	If an ejection system is used, add ejector holes as needed. As with the sprue bushing, undersize the holes by 0.2–0.3 mm (0.008–0.012 in) and ream to size during mold assembly. It is recommended to make sure the holes for the ejector pins will not be too close to the edges. It will weaken the mold especially after reaming.	
Cooling system	Cooling systems will not significantly affect molding cycle times or part quality thanks to the thermal characteristics of PolyJet molds. However, a cooling system can improve tool life; on average, a 20% improvement can be expected. The improvement increases as the depth of the cavity and height of the core decreases since the cooling effects reach more of the surface area of the molding cavity. In recent studies [43], the formable diameter of self-supporting channels has been significantly increased (≥20 mm). A serpentine cooling geometry [44] is able to improve process performance by imposing a cooling curve characterized by a higher slope with respect to traditional channels.	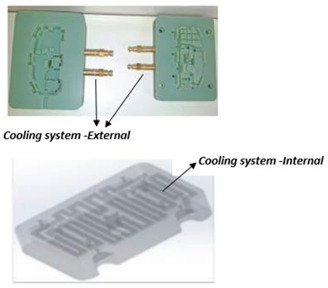 Serpentine cooling channel

**Table 3:** Summary of studies that have evaluated different polymer RTIM processes.

Mold Material	Additive Technique or Machining	Method Used to Evaluate Mold Performance	Injected Polymer	Results (Mold/Part)	Ref.
Aremco 805 epoxy	FDM	Accuracy of injected parts	Polyamide	The dimensional error of the injection-molded part is less than 3%, and the warpage is almost 1 mm across the length of the component.	[59]
Acrylate-based photopolymer	Digital Light Processing (DLP)	Mold failure (# of shots vs. failure)	Liquid silicone rubber	Small-sized parts for drug-releasing (micro)implants were manufactured using micro injection molding. Molds manufactured by DLP did not suffer significant wear when they were used for a low number of microinjection molding cycles (n ~ 8).	[60]
Digital ABS, aluminum	PolyJet	Characterization of molded parts	Isotactic polypropylene	Injected parts showed slower cooling rates in Digital ABS inserts. Parts molded using aluminum tooling did not show a crystal structure. Additionally, parts molded on the digital ABS inserts exhibited higher shrinkage than those molded using aluminum tooling. The change in morphology and the presence of voids significantly affected the tensile behavior of the parts molded in Digital ABS, which broke with little cold drawing and exhibited higher tensile module a higher yield strength.	[61]
PEI (Polyetherimide)	FDM	Thermal performance (specific heat capacity, i.e., Cp, and glass transition temperature); mechanical performance (Young’s modulus, loss factor, and compression tests); structural simulation	Polypropylene, POM	The PEI resulted thermally stable but not suitable for injection molding production of polypropylene parts due to prolonged cooling times and the elastic deformations of the inserts. Regarding the POM parts, the polymer insert did not present relevant damage. However, some problems occurred: difficult de-molding of the POM parts and water permeation through the inserts when the cooling system is active.	[62]
Photopolymer Rigur (RGD450)	PolyJet	Mold failure (# of shots vs. failure) and accuracy of injected parts	Polypropylene	Between 94 to 122 parts (with some geometrical parameters such as undercuts) were injected.	[63]
ABS and nylon (coated with copper)	FDM		Stainless steel powder combined with polypropylene as binder (metal injection molding, MIM)	The heat dissipation of the polymer mold was low compared to that of the metal mold. Therefore, the MIM part needs a longer cooling time inside the mold before ejection.	[64]
Epoxy Biresin, aluminum powder, SL resin, short steel fibers, and tool steel	Mold inserts manufactured by vacuum epoxy casting and stereolithography	Experimental data (pressure, temperature, ejection forces); computer simulation of injection molding (pressure, mold temperature); structural simulation	Polypropylene homopolymer	Molds made by stereolithography are viable if mold temperatures are controlled at 15 °C above the glass transition of the mold material. Otherwise, they exhibit premature failure, and their useful life is not enough for injection molds. The authors estimated the pressure associated with the high shrinkage of the injected polymer on the pins in the mold, which should be taken it into account to avoid failures in these elements.	[52]
Epoxy-acrylate	PolyJet	Experimental data (cavity pressure, strain–time diagram)	Polypropylene homopolymer	The authors implemented on-line monitoring of the cavity pressure during the injection process, and they determined its effect on the deformation of the polymeric inserts by finding a direct relationship between these two variables.	[6]
Different liquid photoresins	SLA 3D printing	Mechanical performance (flexural modulus); thermal performance (heating rate, maximum temperature, heat deflection temperature); cavity dimensions; experimental data (pressure, temperature)	LDPE	It was found that flexural modulus and elongation (two mechanical properties) are more relevant than deflection temperature under load to evaluate the performance of polymer molds made by additive SLA. The latter is useful to produce mold designs that require several changes in shape and dimensions.	[4]
Methacrylic photopolymer	Vat photopolymerisation	Accelerated thermal ageing (weight loss of insert); mold failure (# of shots vs. failure); and mold surface features (average roughness)	-	The application of accelerated thermal aging to polymer mold inserts is a test to evaluate and predict their behavior when they are subjected to thermal loads that determine cyclic stresses. It was found that the stresses induced by the thermal loads of the injection molding process can be reduced by increasing cooling time; however, this produces longer cycle times, thus reducing productivity.	[54]
Ceramic-filled epoxy composite, steel, and aluminum	SLA (polymeric composite)	CAE software (mold temperature); mold failure (# of shots vs. failure); mechanical performance (Young’s modulus, tensile strength, elongation); fiber characterization of polymeric composite	Polypropylene	The reinforced polymer mold withstood the injection of more than 100 parts before failure. The injected parts made of long-fiber reinforced polypropylene showed good mechanical properties and good dimensional accuracy.	[42]
Digital ABS	-	Mold failure (# of shots vs. failure); thermal properties (heat capacity, heat deflection temperature, thermal expansion); CAE software (shear rate, shear stress, mold temperature)	ABS	Failure in the polymer mold was produced by a concentration of high temperatures, especially in the areas of the injection point and the mold cavity because higher shear rates and shear stresses were generated during mold filling. In polymeric molds, the solidified layer in the injected polymer is smaller than in typical steel molds, which indicates a more even cooling of the injected polymer through the flow path.	[3]
Digital ABS, polyamide (PA) 3200 GF, and aluminum	PolyJet, selective laser sintering (SLS), and milling	Surface roughness	Copolymer polypropylene	The parts injected using RTIM showed a lower percentage of elongation compared to those injected in aluminum molds. This is explained by the lower thermal conductivity and higher roughness of the cavities in polymer RTIM.	[7]
Epoxy-based resins	PolyJet	Experimental data (mold temperature distribution, mold temperature vs. time) and mechanical performance (storage modulus, loss factor)	Polypropylene and polylactic acid (PLA)	A comparative evaluation of three types of polymer mold inserts (i.e., without cooling channels, with conventional cooling channels, and with conformal cooling channels) determined that there was no difference in cooling efficiency between the insert without channels and that with conventional channels. The mold insert manufactured with conformal channels reduced the thermal load cycle by up to 70%, with good mold temperature control with respect to the glass transition temperature of the mold material.	[5]
Ceramic photopolymer composite	Vat photopolymerization	Dimensional accuracy (dimensions over 10,000 shots) of mold and part	-	The diameters of the cylindrical elements in the mold were much smaller than the nominal diameters, which was due to the curing process of the photopolymer at the corners and edges after the printing process. The right angles of the corners did not undergo very significant changes in a range from 500 to 1000 injections.	[49]
Digital ABS	PolyJet	Mold failure (# of shots vs. failure); CAE software (injection pressure, injection speed, shot volume, confidence of fill, ejection time, cooling time)	Polycarbonate	Reducing mold temperature and increasing melt temperature were the most important changes to delay failure in polymer rapid tooling inserts.	[57]
Form 2 high temp. resin	Stereolithography, PolyJet	Mold failure (# of shots vs. failure)	Polystyrene	Using a hybrid mold (Master Unit Dye + AM inserts), it was possible to produce up to eighty components using both SLA and PolyJet printed molds.	[65]
Digital ABS, RGD450, Accura Bluestone, Accura SL5530, Accura Xtreme, High Temp, Tough, PerForm, CE221, PA 3200 GF, and steel	PolyJet, stereolithography, CLIP, selective laser sintering, and milling	Mold failure (# of shots vs. failure)	Polypropylene, PA6, and PA6+GF30%	Two polymer reference materials, i.e., PerFrom and PA 3200 GF, offer a great technological advantage to make injection molds because all the polymers under evaluation could be injected without experiencing complete failure of the insert. They can even be used to inject high melting polymer materials.	[1]
Though resin (THO), High Temperature (HT) resins, polyamide 12 filled with 50% of aluminum (PA50Al), and photopolymerization resin (ABS-like)	Stereolithography, laser sintering, and resin photo-polymerization (3D-PolyJet)	Mechanical performance (tensile strength, Charpy impact, flexural strength) and mold failure (# of shots vs. failure)	Elastomeric polyethylene, polypropylene, and ABS	Three mold materials obtained the highest elasticity and flexural modulus: HT, PA50Al, and ABS resin. They are the most appropriate materials to manufacture polypropylene injected prototypes. However, in the injection tests, the prototype mold made of ABS was only able to resist 12 injections before it began to crack.	[66]
Acrylic-based photopolymer	PolyJet	Accuracy of injected parts; thermal properties (specific heat)	High density polyethylene	The injected parts showed large shrinkages. The mold printing material indicates that the glass transition temperature is located at 55 °C.	[67]
VisiJet FTX Green	Stereolithography	Mold failure	Polypropylene	The failure of the insert mold was due to flexural stresses exerted by melt flow on the face of features perpendicular to flow front. Longer cycle times increase the ejection forces that may damage the tool.	[68]
420 stainless steel, bronze alloy, and ABS-like photopolymer	Milling, DMLS, and PolyJet	Thermal properties (specific heat); surface features (average roughness); mold failure (# of shots vs. failure); CAE software (mold temperature, deformation, stress); experimental data (mold temperature); cavity dimensions (average dimensions); mechanical performance (tensile strength)	Polypropylene	Regarding inserts of PolyJet molds, the coefficient of thermal expansion and compressibility of the polymeric insert material should be taken into account to calculate the nominal measurement of the injected part. Additionally, during ejection, ejection force, demolding angle, and cavity surface roughness should be reduced to facilitate ejections with minimal part-to-cavity interference and avoid polymer mold failure. Mold inserts manufactured by DMLS performed similarly to inserts machined from metal, with no failure up to 500 injection cycles.	[41]
Digital ABS, SAE 1045 steel, and Zamak 8	PolyJet and milling	Surface features (average roughness); mold failure (# of shots vs. failure); cavity dimensions (average dimensions)	Polypropylene	The polymer injected in the ABS mold showed a slight increase in tensile strength and elastic modulus, and its impact resistance was increased by more than 30% compared to the parts injected in steel and Zamak. The crystallinity results of the injected polypropylene were not consistent with the cooling rate offered by the ABS mold because said polypropylene showed a lower degree of crystallinity than the parts injected in steel and Zamak.	[69]
Formlabs White Resin, PolyJet Objet RGD515, and PEEK	Stereolithography (SLA), PolyJet, and Fused Deposition Modelling	Accuracy of injected parts	Polylactic acid (PLA)	The molds manufactured by stereolithography and PolyJet produced better finishes on the injected parts, while the mold made of PEEK by molten filament manufacturing presented delamination. In the SLA and PolyJet molds, the accuracy of the injected parts exhibited an average variation of less than 5%.	[70]
Digital ABS, aluminum, and Very High Molecular Weight Polyethylene	PolyJet, milling	Finished mold roughness; finished mold profiles	Cyclic olefin copolymer; polypropylene	The surface finish of 3D printed molds can be improved by applying coatings on the mold surface to inject optical components.	[71]
Photopolymer R11, steel, and aluminum	Stereolithography and milling	Thermal properties (heat capacity); mechanical performance (storage modulus, dimensional change); cavity dimensions (average dimensions); mold failure (# of shots vs. failure)	Polystyrene	The cooling time of polymer rapid tooling inserts is longer than that applied to aluminum and steel inserts due to their higher heat capacity compared to metal inserts. The dimensional changes of the polystyrene moldings (concerning part design and polymeric insert) were in the range 18–4 per cent.	[72]

**Table 4:** Summary of studies into cost models of AM as a disruptive and complementary to IM.

Related Studies	Cost Approach		AM Techniques	Year
	Disruptive	Synergy between AM and IM		
[37]	X		FDM, SLS, SLA	2003
[38,39]	X		SLS	2006–2007
[78]	X		SLS	2012
[12]		X	DLP	2017
[80]	X	X	FDM, POLYJET, SLA, SLS	2017
[79]	X		FDM	2017
[74]		X	DLP	2019
[81]		X	POLYJET	2020
